# A micro-pool model for decision-related signals in visual cortical areas

**DOI:** 10.3389/fncom.2013.00115

**Published:** 2013-08-13

**Authors:** Andrew J. Parker

**Affiliations:** Department of Physiology, Anatomy and Genetics, University of OxfordOxford, UK

**Keywords:** choice probabilities, decision making, visual cortex, perception, connectivity, correlated activity

## Abstract

The study of sensory signaling in the visual cortex has been greatly advanced by the recording of neural activity simultaneously with the performance of a specific psychophysical task. Individual nerve cells may also increase their firing leading up to the particular choice or decision made on a single psychophysical trial. Understanding these signals is important because they have been taken as evidence that a particular nerve cell or group of nerve cells in the cortex is involved in the formation of the perceptual decision ultimately signaled by the organism. However, recent analyses show that the size of a decision-related change in firing in a particular neuron is not a secure basis for concluding anything about the contribution of a single neuron to the formation of a decision: rather the size of the decision-related firing is expected to be dominated by the extent to which the activation of a single neuron is correlated with the firing of the pool of neurons. The critical question becomes what defines membership of a population of neurons. This article presents the proposal that groups of neurons are naturally linked together by their connectivity, which in turn reflects the previous history of sensory stimulations. When a new psychophysical task is performed, a group of neurons relevant to the judgment becomes involved because the firing of some neurons in that group is strongly relevant to the task. This group of neurons is called a micro-pool. This article examines the consequences of such a proposal within the visual nervous system. The main focus is on the signals available from single neurons, but it argued that models of choice-related signals must scale up to larger numbers of neurons because MRI and MEG studies also show evidence of similar choice signals.

Several decades of research have discovered that there are numerous, distinct areas of the cerebral cortex devoted to the analysis of incoming visual information (Zeki, 1993). In all mammalian species so far investigated, there is a primary area of the visual cortex (often referred as V1 for short), which is characterized as receiving the majority of thalamic fibers from the lateral geniculate nucleus (Lund, 1988). Immediately adjacent to V1, and extending some distance anterior to it, is a set of multiple visual areas, collectively known as extra-striate visual cortex. The number of different extrastriate areas is not known definitively but as many as 30 (Felleman and Van Essen, 1991) have been hypothesized in the best-studied species, the macaque monkey. The research agenda for modern visual neuroscience has been very much driven by the goal of searching for and characterizing the distinct and special contributions of these different cortical areas to visual perception and performance (Zeki and Shipp, 1988; Zeki, 1993).

To that end, many recordings of neural signals have been made from the visual cortex of macaque monkeys, whilst they perform a specific visual task for which they have been trained. This combination of recording a neural signal and a behavioral response has been a powerful tool to reveal the specifics of the neural coding of perceptually-relevant signals (Parker and Newsome, 1998). Notably, the goal of understanding the distinct contribution of different visual cortical areas has been advanced considerably by probing the contribution of neural signals to sensory decisions.

## Decision-related activity

Beginning with Hubel and Wiesel (1962), research of the last 50 years has demonstrated in a whole variety of different ways that certain visual cortical neurons have responses that are selective for attributes of the external stimulus. Examples are orientation, direction of motion, chromatic content of the stimulus, binocular depth as well as more complex features of faces, objects and elements of shape perception. Identification of selective responses has been extended beyond simple stimulus attributes to aspects of the visual pattern that reflect its perceptual appearance (see Movshon et al., 1986).

Decision-related activity is distinct from a simple response to an external visual stimulus. Decision-related activity in this context means the identification of specific element of the neural response that is linked to a task-related decision about the stimulus. Thus, in one well-known set of studies, animals were trained to judge the direction of motion of a visual stimulus formed from a group of moving dots (Newsome et al., 1989; Salzman et al., 1990; Britten et al., 1996). Many neurons in the visual cortex are selective for a particular direction of motion of moving dot patterns; some of these neurons also show enhanced response when the animal not only views the visual stimulus but also decides behaviorally that the direction of motion coded by these neurons is the correct choice in the behavioral task.

Data such as these give the appearance that the neuron is not just responding to the arrival of an external stimulus, to which the neuron is selective on account of its pattern of anatomical connections. Rather, these results suggest that a neuron that exhibits a decision-related signal must be a step closer to the cognitive stage of taking a perceptual decision, in comparison with neurons that show no decision-related signals. The suggestion is that such neurons may carry a signal that is related to what the animal is actually perceiving, rather than just reflecting the physiological excitation of the neuron.

Before progressing to such a conclusion, it is important to consider exactly what the basic form of this experimental paradigm is capable of demonstrating. The fundamental result is an association between two events: one event relates to perceptual behavior (the choice), the other is the activation of a neuron. The association is expressed as a change in conditional probability, an increased probability of one event given the occurrence of another. This increase in probability may reflect an underlying causal relationship or may just reflect an increased tendency of both events to happen at the same time, in other words a statistical correlation.

Broadly viewed, there are at least two possible sources for generating a correlation between neural activation and perceptual choices (Britten et al., 1996; Dodd et al., 2001). On the first option, the correlation is driven primarily by fluctuations in the activation of neurons at early stages of visual processing. Noise in the visual pathway results in statistical variation in the activation of neurons during a sequence of trials in which the identical external stimulus is presented on each trial. This noisy variation may be enough to drive the perceptual decision on a fraction of the trials, pushing the decision one way when the activation is strong and favoring the opposite decision when the activation is weaker. In comparing neurons with and without decision-related signals, the ability of noisy variation in a neuron’s activity to influence the perceptual decision may be regarded as evidence that the neurons with decision-related signals are truly on the perceptual pathway for the decision. Although the neurons without decision-related signals may be activated by the external stimulus, the absence of decision-related activity suggests that such neurons are outside the perceptual pathway for the task currently undertaken by the organism.

On the second option, the correlation between neural activation and perceptual choice is driven primarily by the state of the organism leading up to a particular trial. Consider the same sequence of trials just mentioned: if there is an element of pre-judgment or bias about a particular stimulus even before it arrives, this may have the effect of slightly favoring one perceptual choice over the other. If this state of bias is communicated internally through the nervous system to a site where neural activity is being recorded, then the activity at that site may be measurably correlated with perceptual choice. For example, the bias signal may have the effect of enhancing the gain of the sensory response. On this view, the neurons with decision-related signals exhibit them because the brain is putting into action a working hypothesis that these particular neurons are going to be involved in the upcoming perceptual decision. According to this idea, the distinction between neurons with and without decision-related signals reflects the preliminary state of the nervous system before the task is completed on this particular trial. There is a pre-selection of which neurons are most appropriate for the task and consequently those neurons have an enhanced response.

These two possible routes for generation of a choice-related change in neural activity are sometimes referred to as “bottom-up” and “top-down” respectively to give an indication of the likely source of the relationship. However, the real distinction lies in whether the primary cause is regarded as a change in neural activity at the recorded neuron or the behavioral state of the organism. Thus, a “bottom-up” effect generated in some particular set of peripheral nerves may pass into the central nervous system and out again to emerge as a “top-down” effect at another site.

## Decision-related activity and the contribution of neurons to perceptual decisions

A reason for the interest in decision-related activity is that it has been widely presumed that neurons with this kind of activity may be playing a special role in the supporting perceptual decisions. It was always recognized that the relationship between neural events and perceptual events, as measured with this approach, is fundamentally a correlation rather than a proven causal relationship. Nonetheless, the presumption was that neurons with decision-related activity must at least be candidates for a direct involvement in perception. This was advanced as a general principle by Parker and Newsome (1998): “Fluctuations in the firing of some set of the candidate neurons to the repeated presentation of identical external stimuli should be predictive of the observer’s judgment on individual stimulus presentations.” This statement should not be viewed as a direct and definitive test of the involvement of a set of neurons in a perceptual decision. The principle articulates the argument that in the absence of evidence even for a correlative link, then there is in reality no case for pursuing other lines of investigation to determine whether there is a perceptual role for some set of neurons. This is true even if the neurons are exquisitely sensitive to the visual stimuli to be discriminated or detected. The correlative link is therefore a weak test for the role of neurons in perception but it can nonetheless be advanced as an important minimal requirement.

## Size, specificity and prevalence of decision-related activity

As well as noting enhancements of firing rates in certain neurons, decision-related activity has been assessed statistically; for a recent account, see Nienborg et al. (2012). The standard approach has set up the following test for the presence of decision-related activity. Consider a set of trials upon which the activity from a single neuron or small group of neurons has been recorded in response to repeated presentations of the same stimulus. When these responses to repeated presentations are considered, it may be the case that the behavioral response is different on some of those trials in comparison with others. When the behavioral response is a binary choice, the trials then simply divide into two types according to the binary choice on that particular trial. The statistical assessment of choice-related activity is to ask with what reliability could one predict the behavioral choice at the end of the trial purely on the basis of neural responses. In other words, if an external observer of the trials knows the statistical distribution of activity of the neuron for different trial outcomes, what are the chances of correctly predicting the monkey’s decision on this particular trial, given only the activity of the neuron on this trial?

Clearly, if there is no decision-related activity arising from the neuron, then the external observer’s chances of correctly predicting a binary choice judgment are 50:50. If the neural activity has a decision-related component, the external observer’s chances rise: a neuron that is completely reflecting the sensory decision will have a complete separation in the statistical distribution of activity associated with each of the two choices.

Consider a simple decision, which is a binary choice about direction of movement (as in the first clear demonstrations of these phenomena). If the experimenter is recording from a sensory neuron that has a preference for movement of the visual stimulus to the left as opposed to movement to the right, then decision-related activity is normally expected to be an enhancement of firing when the behavioral decision is a choice for leftwards movement. It might be thought that recording from a motor neuron right at the output stage could fulfill this specification for decision-related activity. This misses the point that the above logic really applies to a sensory neuron with a particular stimulus preference. The way that the choice is recorded does not need to be specified, but the ability to identify the stimulus selectivity of the neuron is critical. Only with this identification can the behavioral responses be classified as with or against the sensory properties of the neuron under study. Thus, in the original experiments, when a decision was made that a visual stimulus was moving to the left, the activity was enhanced in neurons that preferred leftwards moving stimuli as against rightward moving stimuli.

It is usual practice to evaluate decision-related firing with stimuli that only have a zero or weak movement in a particular direction: this allows a number of mistakes to arise in the set of behavioral choices over a sequence of trials. Without this variability of the behavior, it would be impossible to measure choice-related activity from the neuron. This reflects again that the fundamental measure of decision-related activity is a correlation between two types of events: sensory decisions and neuronal activity. At first thought, it may seem remarkable that any correlation could be detected. There are many neurons available for recording, yet only one is chosen by the experimenter, typically by adjusting the position of the electrode within the cortical grey matter until a clear action potential is isolated. Considering that, in a volume of tissue approximately the size of a cortical column (1 × 1 × 2 mm), there may be as many as 100,000–150,000 neurons (Rockel et al., 1980; Carlo and Stevens, 2013), it might be thought improbable that the one encountered by the experimenter is also the neuron that is correlated with the decision. Yet, for at least some of these recordings, correlations between neural firing and decision have been established.

Furthermore, for some individual studies, the correlations are fairly tight, with choice probabilities as high as 0.67–0.75 (Dodd et al., 2001; Ghose and Harrison, 2009). Under the (incorrect) assumption that the firing of neurons is independent, there would be a striking consequence. Given a choice probability of *P*, the probability of not correctly predicting the outcome of the decision is (1 − *P*); with *N* independent neurons, this probability of incorrect prediction falls to (1 − *P*)*^N^*. With this simple multiplication of probabilities, the probability of failing to predict decision correctly is simply 1 − (1 − *P*)*^N^*, so that the activity from as few as three neurons would be sufficient to give a statistically adequate (better than 95% correct) prediction of the decision on an individual trial. The improbability of encountering those three neurons among 150,000 is self-evident. More typical values reported for choice probability are 0.55 on average (Britten et al., 1996), which might suggest the availability of about five relevant neurons on the same calculation, but even this adjustment barely scratches the surface of the problem.

It was immediately appreciated that the estimate is improbable because the assumption that the responses of the neurons are statistically independent is incorrect. Direct measurements of the correlation between the firing of single neurons during the motion detection task have suggested values of 0.15–0.18 (Zohary et al., 1994). A more broad-ranging set of cases is summarized in Cohen and Kohn (2011). Correlations of this level are sufficient to change model-based estimates of the pool size for the decision, raising it to 50–100 neurons (Shadlen et al., 1996). By contrast, it has been argued that the time-course for assessment of choice probabilities is critical in accepting this conclusion. In a task in which a single sensory event must be detected in a reaction-time paradigm, neuronal signals discriminating correct-detects from failures-to-detect are strongly separated, as are signals from the same neurons that discriminate correct-detection from false-positives (Ghose and Harrison, 2009).

Recently, neuronal sensitivity and choice probability in the central nervous system have been compared at cortical and sub-cortical sites involved in a judgment of heading direction for which the primary sensory signal is vestibular in origin (Liu et al., 2013). These authors report differences in the correlation structure of noise at these different sites, which they relate theoretically to the different sizes of choice probability observed at those sites.

## Top-down signals

**Figure 1:**
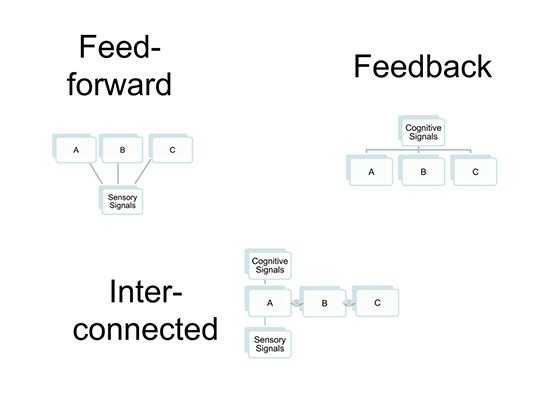
**Three connection patterns that lead to measurable choice probabilities in neurons A, B and C**. In the feed forward model, the three neurons receive common input from a site providing sensory signals. The common driving of A, B and C is sufficient to generate measureable correlation if two neurons are recorded simultaneously. In the feedback model, all three neurons receive a feedback signal in common from a more central site, with the same consequence for measureable correlations. In the interconnected case, only neuron A receives the feedforward or feedback signal, but neurons B and C exhibit signs of its arrival because they are connected to A, which influences their activity and generates correlations between all three neurons.

The above discussion is fundamentally based upon a view of decision-related neural activity that regards the underlying causal link as originating with the sensory neuron: a noisy fluctuation in neural activity tips the decision one way or the other on a particular trial, with the result that a correlation between neural activity and perceptual choice (see Figure 1 “Feedforward’’). On this view, the neuronal activity in the sensory neuron has two components: one is related to the visual stimulation, another arises from noisy fluctuations intrinsic to the nervous system.

An entirely distinct view of choice probability has been entertained from the earliest papers. On this view, there are also two relevant sources of input to the neuronal activity: one arises from the visual stimulation as before, the other path of influence on the recorded neuron reflects the cognitive state of the organism just prior to the trial that is being tested (Krug, 2004). The cognitive state is manifest as a bias for one perceptual choice rather than another. Such a bias signal is thought to be targeted at neurons that prefer a visual stimulus that corresponds to the perceptual choice favored by the bias state. The state of bias may be set by factors such as the history of reward or failure, the diet of visual stimulation or as yet unidentified fluctuations in internal state.

In this case, the causal direction is different. The physiological correlate is a change in the firing of certain neurons elsewhere in the brain: these neurons communicate, possibly indirectly, with the sensory neurons that are being studied by the experimenter, forming what is often referred to a “top-down” signal (see Figure 1 “Feedback’’). As internal state changes, under the influence of reward conditions, attention or stimulus history, this change affects both the organism’s choice on the upcoming trial and the firing of the neuron that is being recorded. The initial study on choice probability carefully considered and rejected the notion that all aspects of choice-related firing could be explained by variations in attentional state (Britten et al., 1996). The experimenters pointed to differences between the firing patterns induced by modulation of attention and those related to perceptual choice. Later work has established that at least a component of the choice-related firing may be driven by “top-down” factors.

The first piece of evidence is negative. Shadlen and colleagues carefully modeled the noise-driven, “bottom-up” route for the generation of choice related firing. Within a population of model neurons, whose firing patterns were closely matching the statistical characteristics of the recordings of V5/MT neurons, Shadlen et al. (1996) explored the conditions under which a realistic combination of parameters could be achieved to explain two parameters of neuronal performance. The first parameter was the choice probability already described; the second parameter was the detection performance of the population of neurons, when their individual signals were pooled to predict the behavioral performance of the monkey. Within a range of values compatible with observed values of the detection performance of single neurons and interneuronal correlations measured during performance of the motion-coherence detection task, it was impossible to accommodate choice probabilities as large as 0.67 as found in some studies. The model with parameters similar to those considered most likely by Shadlen et al. (1996) leads to small numbers for the pool size of neurons (less than 10), which implies a return of all the issues for finding and identifying the relevant neurons. This analysis is specific to the conditions discussed and explored: in particular, with a change in the interneuronal correlation, it may become possible to find a set of model parameters that allows all possibilities.

The second piece of evidence is more direct. Nienborg and Cumming (2009) examined the performance of disparity-sensitive neurons in the secondary visual area of V2 of macaque monkeys. They were following up an earlier observation of theirs (Nienborg and Cumming, 2006), in which they found that V2 neurons exhibited significant choice probabilities with a disparity-detection task, while V1 neurons did not. In the follow-up experiment (Nienborg and Cumming, 2009), they introduced a noisy signal within the visual stimulus within each individual trial: they arranged for a two-second presentation of a stimulus trial to be broken down into eight separate epochs, during each of which a modest fluctuation of disparity, positive or negative, was superimposed upon the programmed disparity for the trial. The strategy was to examine whether these imposed fluctuations of stimulus disparity were manifest as noise in the firing pattern of the neuron and in particular to ask whether the imposed fluctuations had similar or different effects upon the neuron and the monkey’s behavior. If the effect of imposed fluctuations were very similar on neuron and behavior, then there would be a strong inference that the choice probability is fundamentally an early, “bottom-up”-driven process. On the other hand, a marked difference in the effects of the inserted noise on the neural and behavioral measures on a trial-by-trial basis would strongly suggest the presence of an additional signal, potentially from a “top-down” source. Nienborg and Cumming’s results and analysis favored the second of these outcomes and therefore strongly suggest the inadequacy of a simple account of choice probability based on noise early in the visual processing.

## Choice probabilities on a coarser scale of granularity

Choice probabilities have also been investigated with methods that do not involve the recording of single neurons. In one case, brain regions that encode the presentation of faces with expressions of fear were distinguished in an MRI study from brain regions that encode the presentation of faces showing expressions of disgust (Thielscher and Pessoa, 2007). A single trial fMRI study was then conducted using signals from the two brain regions classified according to the behavioral response of the participant to a perceptually ambiguous expression. Calculated choice probabilities for these brain regions were very close to the values of 0.55 reported from the initial single neuron studies in macaque cortex.

Two points are relevant to bear in mind about these results. First, the identification and selection of ROIs for the MRI analysis is dependent in part on identification of clusters of responding voxels. This step in the analysis is likely to mean that the each ROI consists of a set of voxels with correlations in the activity between voxels in the cluster. This is somewhat similar to the circumstances in which single neurons’ responses are correlated. Second, the stimulus-related responses of the MRI clusters are less reliable than expected and less reliable than many single neurons in the macaque’s brain. Correct identification (stimulus probability) of the fear vs. disgust an expression of the faces was at best 0.69, this being under circumstances in which the behavioral judgments reached 1.0. This leaves some explanatory gap for the MRI data, in terms of a complete account of the behavioral performance, and raises the issue of what values would be achieved for choice probability if the precision of the MRI signal were greater.

In a separate study, a single trial analysis of EEG data was used to identify choice-related brain activations during a task to discriminate faces from automobiles (Philiastides and Sajda, 2006). In this case, the combined measurement of neural and psychophysical performance showed that the neural signals had the precision to extend over the full range of human behavioral responses. The early phase of the EEG signal gave inconsistent results in the choice probability analysis: only three out of six participants exhibited significant choice-related neural responding. In the later phase of the EEG signal, robust evidence for choice probabilities emerged, with values between 0.61 and 0.81. The late phase of the response studied here arises between 300 and 450 ms after stimulus onset (depending on the individual subjects’ responses). There is therefore the possibility that some of the choice-related differentiation of this signal is related to post-decisional processes. It is also worth comparing this point with the single-neuron data in sensory areas, which typically shows some evidence of decision-related signal as soon as stimulus onset occurs.

Data of this kind demonstrate that the phenomena identified in single neuron recording experiments with macaque monkeys have parallels with observations using non-invasive measures, which can be achieved with human volunteer participants. However, thus far, the parallels are not exact. These experimental approaches also raise interesting issues of scale in brain measurements. Neurophysiologists typically spend a great deal of planning and effort to target single neurons with specifically interesting signals relating to cognitive behavior. It remains unclear how a local group of neurons might generate a choice-related signal that would be detectable on a larger scale of measurement, some tens of mm^3^ of tissue in size. Clearly, the role of correlated activity across a set of neurons must be important in generating a scale-independent regularity of this kind. Often however the signals from small groups of neurons will fall below the resolution level of these non-invasive techniques, so the choice-related signals may be visible only under highly specific experimental paradigms.

## Interpretation of choice probabilities

A major motivator remains to understand what do these set of results tell us about the role of neurons (or other neural structures) in perception? If the early-noise model is correct, are we allowed to conclude that neurons with choice probabilities are indeed on the perceptual pathway: that is, in general, does the activity within neurons that show significant choice probabilities control the perceptual decision by comparison with similar activity in neurons with no significant choice probability? If the “top-down” view is correct, is it correct to presume that the top-down signal is directed specifically to the neurons relevant for the perceptual decision; or does the cognitive signal leak out to influence the firing of neurons that actually have no contribution to the task?

A recent theoretical analysis casts some light on this question. Haefner et al. (2013) examined the role of interneuron correlation on choice-related signals in a neural population: rather than considering the distributed activity of a population of neurons, they considered the mean behavior of a large population. With this analysis, it became clear that when a neuron exhibits a choice probability, even a large one, this tells us more about which other neurons this recorded neuron is connected to, rather than revealing the presence of a subgroup neurons that are indeed on the perceptual pathway (see Figure 1 “Interconnected’’).

**Figure 2:**
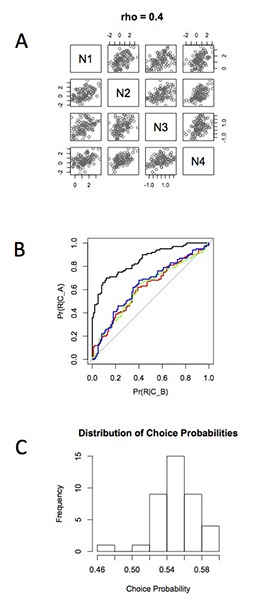
**Illustrating the effects of correlation on neuronal signals in a population. This simulates a network such as that in Figure 1**. **(A)** Four neurons arranged to have an interneuronal correlation (rho) equal to 0.4. Neurons 2 to 4 are simulated as zero mean, unit variance Gaussian random variables. Neuron 1 is the same, except an artificial choice signal (value = 1.5) is inserted. **(B)** The responses of the four neurons are subjected to ROC analysis to reveal the choice related signal. The black curve projecting up to the upper left corner represents the detectability of the choice signal on Neuron 1. The colored curves are for Neurons 2,3,4. These neurons received no choice signal in themselves but acquired the choice signal by virtue of their correlated variance with neuron 1. **(C)** Summary histogram of choice probabilities induced in population of 40 neurons by choice signal inserted into single neuron and presence of interneuronal correlations inducing the choice probability in the other 39. Estimate of 100 repeated trials with a choice signal of 1.5 as at **Figure 2**.

This principle is illustrated in the simulation illustrated in Figure 2. A small set of neurons was simulated with Gaussian random variables. To just one of these neurons, a choice signal was added—in this case by adding an additional level of activity to that neuron, thereby simulating the effect of a single top-down signal targeting just one neuron in the pool. The pool of neurons is interconnected, thereby generating correlated activity in that pool (Bair et al., 2001).

Whether or not a neuron in involved in a perceptual decision is governed by a set of weights, one for each neuron that defines the contribution of that neuron to the decision variable. Thus for a particular decision in favor of an outcome, the decision variable is formed by a weighted sum of the activations of a population of neurons. The authors refer to this process as “reading-out” a decision from a profile of activity across a population of neurons and these weights are often referred to as the “read-out weights’’. The weights for a particular decision are stable from trial to trial of the decision task but the weights may be gradually adjusted over time under feedback about the outcome of decisions already made.

An important question is therefore what consequences for measured correlations are generated by the pattern of neural connectivities? Correlations among a group of multi-variate entities are represented by the correlation-matrix. This matrix is always symmetric since the correlation *C* between elements *i* and *j* are always constrained by *C_ij_* = *C_ji_*.

An equivalent matrix that defines the connectivity between a pair of neurons *i* and *j* does not have this constraint. For example, with a simple feed-forward connection, the firing of neuron *i* may influence neuron *j* without there being an equivalent influence from *j* to *i*. Another complexity of neuronal relationships that is not captured by the correlation of firing between two neurons is the combination of excitatory and inhibitory influences; another issue is the difference between long-range excitatory connections and short-range inhibitory connections that characterizes cortical connections as revealed histologically.

Nonetheless, it is now demonstrated that in theoretical models that the structure of neuronal connectivities brings about distinct patterns of correlation and, more encouragingly, the observed pattern of correlations provides some insight into and constraints upon the underlying pattern of connectivities (Kriener et al., 2009; Pernice et al., 2011). Indeed, Haefner et al. (2013) suggest some ways in which structure within the correlation matrix can be exploited to recover information about which neurons are contributing directly to the perceptual decision. Empirically, Liu et al. (2013) compared different sites and successfully related the differences in choice probability to measurable differences in noise correlations between those sites. However, comparison of this kind do not address the more fundamental question of what conclusions are justifiable based on the observation of statistically significant choice probabilities at a brain site.

Another route into interpreting the pattern of correlated activity is to bring in the time domain. Various attempts have been made in neuroscience to base the analysis of correlations on the concept of Granger causality: see Seth (2008) for review. This asserts that if the variation in signal in a structure *A* at time *t* is *A(t)*, then a signal in structure *B* at time *t-δ* explains what is happening at *A*, if *A(t)* is correlated with *B(t-δ).* Of course, this situation can be created if two signals leave a third source *C* and arrive at both *A* and *B*, but there is a relative delay in transmitting the signal from *C* to *B* in comparison with from *C* to *A*.

The conclusion from these various issues is that patterns of correlation may provide some interesting constraints on the set of possible connectivities between groups of neurons. Nonetheless, the same pattern of correlations may be generated by quite different underlying connectivities (Pernice et al., 2011). In the future, it will be necessary add in information from other sources of evidence, separate from what can be learnt from direct measurement of correlations. The obvious next step is to couple measurements of correlated activity with some method of intervening in the functional signaling among the group of neurons, such as molecular activation or silencing, or with some separate and independent line of evidence about the role of the group of neurons in perceptual decisions, as attempted by Krug (2004).

## Alternative checks on the functional significance of choice probabilities

Given the complexities of interpretation raised by the presence of interneuronal correlations in groups of neurons, it seems reasonable to ask the question whether we can gain an independent assessment of the functional significance of observing a statistically valid choice probability within the activity of a single neuron. If, as seems clear, the identification of decision-related firing does no more than present circumstantial evidence that the neuron might be a candidate for involvement in the perceptual decision, it seems worthwhile to consider alternative routes for validating the neuron-perception link.

Two experimental strategies present themselves. First, it might be possible to identify a neuron that has a choice probability and directly test the consequences of activating that neuron for the perceptual choices made by the participant. Second, it might be possible to generate a corroborative test of the role of neurons that have already been identified as carrying a choice probability. To my knowledge, only the second of these tests has been attempted.

Recording in V5/MT during the performance of a stereo/motion task by macaque monkeys (Krug et al., 2004) tested single neurons for the presence of choice probabilities during the performance of the task, which required a response to the sign of binocular depth in a structure-from-motion stimulus, to judge its direction of three-dimensional rotation. The same neurons were also tested to see whether there was a consistent response to the stereo disparity of binocularly anti-correlated stimuli. The authors confirmed that changing the disparity of these binocularly anti-correlated stimuli failed to yield a coherent change in the depth percept. Hence, by this test neurons that carry a perceptual signal ought not to respond to changes in the disparity of binocularly anti-correlated stimuli. Taking each test alone, the authors identified V5/MT neurons that carry signals that are congruent with the perceptual effects. However, on comparing tests, there was no fixed pool of neurons, whose activation would lead to a unitary account for the binocular depth percept. Specifically in the context of this discussion, the authors found that excitation of neurons that are proven to have a statistically measurable choice probability does not necessarily lead to a change in perception.

This result places a clear challenge to the interpretation of decision-related firing in single neurons. At least some neurons may exhibit decision-related signals in a perceptual task, in cases when there is clear alternative evidence that their activity is unrelated to perceptual decisions. There are a number of ways of explaining this complexity but in the rest of this article, I focus on the role of neuronal connectivities in generating results of this kind.

## Irrelevant neurons in the pool?

Up to this point, the discussion has been essentially neutral on the issue of how connections between neurons arise. Although functionally open connections between neurons will always have consequences for the size of measurable correlations in their firing, it is possible to identify a number of distinct ways in which correlation may be generated. The classic “bottom-up; top-down” distinctions have been mentioned already. In the bottom-up approach, interneuronal correlation is generated by trial-by-trial fluctuations in the strength of signals arriving from earlier sensory stages. These signals pass into a group of neurons, sending a common signal that is stronger on some trials and weaker on others. This variation is transmitted in parallel to this group of neurons, which will therefore exhibit measurable interneuronal correlations regardless of whether the members of this group are interconnected with each other. A similar picture applies for top-down signals. Here a signal may arrive in a group of neurons from another cortical site, typically conceived as sending a cognitive signal relating to attention or other task-related. The effect is very much the same as before. Trial-to-trial variations in this signal may be transmitted in parallel to the group of neurons that the experimenter has under measurement; again these variations will generate interneuronal correlations in firing among the group; as before there is no requirement for the members of the group to be functionally connected to each other to generate correlations among them.

Neither of these schemes is particularly realistic when considered against the known facts of cortical connectivity. Neurons that are receiving in common sensory inputs or source of cognitive influence are likely to be connected among themselves, rather than isolated from each other. These interconnections are also a mechanism by which interneuronal correlations can be generated. With this in mind, it is possible to hypothesize a third route by which neurons may acquire choice-related firing during the performance of a perceptual task. This is through the so-called “innocent bystander” route; in this case, a neuron is not actually part of the decision process but it is functionally connected with neurons that are involved in the decision. By sitting as a member of a network that has functional connections with neurons that are directly involved in the decision, the “innocent bystander” acquires measurable signals relating to the decision itself (see for example, Cohen and Newsome, 2009). In cognitive terms, these signals represent knowledge without responsibility: the signals are present and could be read out to learn about the decision, but they don’t actually affect the decision one way or the other. In mechanistic terms (Haefner et al., 2013), this relationship is expressed as neurons that have a measurable interneuronal correlation but a zero weight in the decision.

The pure “innocent bystander” model, which is illustrated in Figure 1 “Interconnected’’, is neutral about how and why the interconnections between neurons arise. One route is to argue that these interconnections have no functional purpose at all. They are just accidental. On this approach, it is possible to take a minimalist view of the significance of measuring a choice probability in a particular neuron. As noted earlier, the presence of the choice probability simply indicates no more than the haphazard association of the recorded neuron with some other neuron, which is the true conveyor of information forming the decision. This minimalist view is useful for stripping away assumptions about what a measurable choice probability tells us about the role of a neuron in perceptual decisions. However, it is not a very realistic view about the organization of the nervous system.

The suggestion here is the fairly conventional view that connections between neurons build up and are refined as a consequence of developmental process followed by activity-dependent tuning of neural connections. Granted that there may be some random and haphazard connections, but the idea that this is the dominant mode of connectivity is far-fetched and implausible. What is more consistent with knowledge about the structural and functional connectivity of the cortex is to acknowledge the following. Whenever a new study of a perceptual task is begun, the participants (whether human or non-human) arrive at the beginning of the task with a highly structured visual nervous system. The learning of the specific task required by the experimenter is overlaid on this past experience. That previous experience will have shaped and adjusted the visual nervous system, creating sets of connections that are relevant for the range of tasks involved in everyday living plus any specific tasks for which the experimenter may have been trained. Thus, neurons are already organized into groups and functional circuits before the experimental study begins.

When a new psychophysical task is performed, a group of neurons relevant to the judgment becomes involved because the firing of some neurons in that group is strongly relevant to the task. This group of neurons is called a micro-pool. It is suggested that the selection process for forming a decision pool relevant to the task is by selection of a micro-pool not by selection of individual neurons. Hence, some neurons in the micro-pool become associated with the performance of the task on the basis that they are connected with the neurons that are firing strongly in a task-relevant way. These other neurons have firing that is correlated with the activity of the relevant neurons, largely because past experience has shaped the present state of the nervous system, equipping it with neural circuits or cell assemblies that have been functionally useful. Over time the membership of the micro-pool may be adjusted as a result of perceptual learning. But there will generally be some inefficiency and therefore there will always be some irrelevant neurons in the micro-pool, whose firing is partially correlated with the firing of neurons relevant to the task. On this view the connections within the pool are not generated by random processes but are present for functional reasons that happen to be only partially relevant to the task being studied.

## Choice probability as an index of functional connectivity?

Whether we regard choice probabilities as arising from a bottom-up or top-down source, it is clear that at a particular cortical site, where neurophysiological recordings are being conducted, a signal enters among a connected group of neurons. This signal propagates among that restricted group. We assume here that there is no distinction in the way that the choice-related signal propagates from neuron to neuron and the way that other signals propagate among the same neuronal group. This is a standard baseline assumption: it implies that all signals from one neuron to the next are treated equivalently, simply passing from one neuron to the other with a signal strength governing by a single value for the synaptic weighting from neuron *A* to neuron *B*. Other assumptions are possible but they would involve separate signaling pathways exiting the neuron, with one of the pathways preferentially channeling the choice-related signal and the other channeling the pure stimulus-evoked signals.

**Figure 3:**
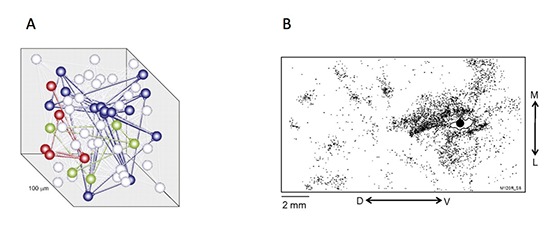
**Two experimentally observed measurements of the interconnectedness of pools of neurons sufficient to generate interneuronal correlations**. **(A)** Shows connectedness on a scale of the order of 100 μm (Perin et al., 2011), while **(B)** shows connections on a much larger scale that traverse a large fraction of cortical area V5/MT in the macaque (Ahmed et al., 2012).

Under this assumption, once a choice-related signal has entered a local network of cortical neurons, it is able to propagate through the network using the same synaptic connections that deliver other signals (Bair et al., 2001). Recent recordings from multiple neurons in cortical tissue slices have used photic or electrical stimulation to probe the functional connectivity between small groups of neurons (Yoshimura et al., 2005; Perin et al., 2011). The connectivity revealed by these experiments suggests the interlinking of groups of neurons (chiefly pyramidal neurons from sensory neocortex), with a specialized linking of some dozens of neurons over 100–300 μm distances in cortical tissue. Neurons forming a single cluster are individually spaced such that it is easy to embed multiple clusters within a single compartment of cortical tissue. An arrangement such as this means that there are favored paths of connectivity throughout a single block of cortical tissue (see Figure 3).

An indirect line of support for this view comes from the work of Ghose’s lab (Ghose, 2006; Ghose and Harrison, 2009). In these studies, the animal is presented with a pair of arrays (each about 5–7 degrees in visual angle), composed of 31 Gabor stimulus elements, each one moving back and forwards according to independent random sequences. Within this stream of random sequences, a brief pulse of coherent motion is introduced to one of the two arrays, during which elements move consistently in a single direction. The animals’ task is to detect this motion pulse, responding with an eye movement. As mentioned earlier, the choice probabilities calculated for V5/MT neurons during this task are substantially higher (0.64) than those found for other motion detection tasks in the same visual area (Ghose and Harrison, 2009). The reasons advanced in that paper for the larger choice probability relate to the temporal precision of the information that must be processed for this sensory task.

An alternative view, not necessarily incompatible with that of the authors, is that points across the cortical V5/MT representation of the array of Gabor stimulus elements are likely to be served by neurons connected on the basis of similar motion preferences. Applying a coherent pulse of motion across the stimulus array consistently drives this strongly connected network of neurons. This may be distinguished from the random-dot kinetogram stimulus, in which the relevant motion signal is distributed at inconsistent locations across the field of random dots. Thus, it is true that the kinetogram differs from the array of Gabors because it provides a temporally brief pulse of motion. However the array of Gabors also delivers a pattern of motion that spatially coherent and also is presented with low spatial uncertainty (Davis, 1981; Nachmias, 2002; Bach and Dolan, 2012) since each motion pulse is introduced into the display at the spatial location of a highly visible stimulus element.

In regard to the connections between neurons, the organization of a network that responds to coherent motion across spatial positions is something that can be potentially constructed on the basis of daily experience outside the task-specific training that the animals participating in this study will have received. A similar consideration applies to the rotating cylinder stimulus, for which high choice probabilities have been reported (Dodd et al., 2001). This stimulus delivers a combination of motion parallax and binocular depth that is often experienced by a moving observer, who is translating in the world and simultaneously counter-rotating the eyes to maintain gaze on a static point in the visual scene (Krug and Parker, 2011). By contrast, the knowledge about the spatial uncertainty attached to a particular stimulus is something that can only be learned in a task-specific manner but Ghose (2006) has demonstrated significant learning effects in his experimental paradigm. These learning-related changes are presumably manifest within the connectivity matrix of sensory neurons.

The potential implication for the analysis of choice probabilities is that the correlation matrix, which is related to the connectivity matrix, is far from uniform in its set of values. Some connections will be strong, implying relatively high correlation values for the pair of neurons concerned, whilst other connections will be weaker, resulting in lower correlation values. For the neural tissue of V5/MT, in which many experimental observations of choice probability have been made, the pattern of intrinsic connections within the cortical area is critical. A recent study (Ahmed et al., 2012) has revealed an internal organization of projections within V5/MT extending as broadly as 10 mm across the cortical surface with an approximately 2 mm repeat pattern. It would be of considerable interest to measure the interneuronal correlations and decision-related activity between cortical sites across this repeating pattern.

## Conclusion: a micro-pool model

This article has analyzed the behavior of three different models of the generation of decision-related firing, particularly its manifestation as choice probability during the performance of a visual task. These different models were chosen primarily to illustrate their individual shortcomings and to demonstrate that the truth must lie somewhere between these. A second theme of this article is to consider the structure of interneuronal correlations as a potential influence on the generation of choice probabilities. It is concluded that the clustering of neurons into functional groups almost certainly precedes the training for particular perceptual tasks. The initial selection of neural mechanisms suitable for task performance is probably at the level of these functional groups, which were termed here a “micro-pool’’. The numerical size of the pool is uncertain at this stage: it may arise from small clusters of tightly packed neurons or it may spread over mm of cortex by long-range connections. Two important factors that determine the choice probability measurable from a micro-pool are the size of the choice signal arriving with one or more neurons in the pool and the interconnectedness of the pool itself.

It is concluded that the clustering of neurons into functional groups almost certainly often precedes the training for particular perceptual tasks. The initial selection of neural mechanisms suitable for task performance is probably at the level of these functional groups, which were termed here a “micro-pool’’. The size of the pool size is uncertain at this stage and may vary from task to task: it may arise from small clusters of tightly packed neurons or it may spread over mm of cortex by long-range connections. Two important factors that determine the choice probability measurable from a micro-pool are the size of the choice signal arriving with one or more neurons in the pool and the interconnectedness of the pool itself. It is concluded that the generation of strong choice probabilities seen in some studies (Dodd et al., 2001) may reflect a selection process, in which the dominant structures that emerge to control perceptual behavior are tightly interconnected groups of neurons. With this organization, it is possible to generate a sufficiently high correlation among activities of a group of neurons, some of which are the primary processing units for the choice-related signals.

## Conflict of interest statement

The authors declare that the research was conducted in the absence of any commercial or financial relationships that could be construed as a potential conflict of interest.
